# Prognostic Significance of Visit-to-Visit Ultrafiltration Volume Variability in Hemodialysis Patients

**DOI:** 10.3390/biomedicines13030717

**Published:** 2025-03-14

**Authors:** Balázs Sági, Tibor Vas, Éva Fejes, Botond Csiky

**Affiliations:** 1 Nephrology and Diabetes Center, 2nd Department of Internal Medicine, Clinical Center, University of Pécs, 7624 Pécs, Hungary; vas.tibor@pte.hu (T.V.); botond.csiky@gmail.com (B.C.); 2 National Dialysis Center Pécs, 7624 Pécs, Hungary; 3 Hospital of Komló, Clinical Center, University of Pécs, 7623 Pécs, Hungary; efchengirl@gmail.com

**Keywords:** end-stage kidney disease, hemodialysis, ultrafiltration volume variability, survival, MACE, echocardiography

## Abstract

**Introduction:** Patients on chronic hemodialysis (HD) have significantly higher mortality compared with the general population. Cardiovascular (CV) disease is the primary reason for death in these patients. Suboptimal extracellular fluid management increases the CV risk of HD patients. We aimed to study the effect of visit-to-visit ultrafiltration volume (UV) variability on CV events and mortality in chronic HD patients. **Patients and Methods:** In our study, 173 chronic HD patients were included (median age: 63 ± 13 years; 53% men). Ultrafiltration volume (UV) variability was analyzed retrospectively for 24 months. The standard deviation (SD) and coefficient of variation (CV) were calculated using the indices of UV variability. CV is the SD divided by the mean. The obtained parameters were SD and CV of the UV: UVSD and UVCV. UV data during the observation period were recorded and used to calculate UV variability. Routine transthoracal echocardiography was performed. **Results:** Patients were divided into groups based on the median of UVSD, low-UVSD (<568 mL) and high-UVSD (≥568 mL) group; and also based on the median of UVCV, low- (<0.29) and high-UVCV (≥0.29) group. All-cause mortality was significantly higher in the high compared to the low-UVSD (21/84 vs. 9/89; *p* < 0.001) group. Similarly, mortality was higher in the high-UVCV group compared to the low-UVCV group (18/78 vs. 12/95; *p* = 0.005) after 24 months. Major adverse CV event (MACE) rates were also significantly higher in the high- compared to the low-UVSD group (20/84 vs. 8/89; *p* < 0.001). Similarly, the MACE rate was significantly higher in the high-UVCV group compared to the low-UVCV group (15/78 vs. 13/95; *p* = 0.029) after 24 months. There was no significant difference between the groups in CV mortality. UVSD correlated with parathormone (PTH) level (r = 0.416; *p* = 0.015), and UVCV with total cholesterol (r = 0.419; *p* = 0.015). Left ventricular end-diastolic diameter (LVEDD) and end-systolic diameter (LVESD) were higher in the high-UVCV group compared to the low-UVCV group (49.95 vs. 52.08; *p* = 0.013 and 32.19 vs. 34.13; *p* = 0.034). **Conclusions:** According to our results, high UVSD and UVCD are associated with increased all-cause mortality and MACE rates but not CV mortality in chronic HD patients. Cardiovascular changes caused by increased UF volume variability during HD may contribute to higher CV morbidity and mortality in these patients.

## 1. Introduction

Patients on chronic hemodialysis (HD) have significantly higher mortality compared with the general population [1,2]. Cardiovascular (CV) disease is the primary reason for death in these patients, being responsible for over half of all mortalities [3,4]. Exploring risk factors is crucial for enhancing the survival rate of these patients. Volume management is an essential and vital component of dialysis treatment [5]. Increased risk of cardiovascular mortality in HD patients is linked to extracellular volume overload [6,7]. However, intradialytic hypotension (IDH) caused by aggressive volume removal, resulting in myocardial stunning, is also associated with higher mortality and morbidity [8]. At present, we do not have exact markers for volume assessment, leading to uncertainties in obtaining optimal volume balance in HD patients. In clinical practice, determining dry weight is subjective and can be impacted by various factors [9]. The challenge is determining the volume status and the appropriate amount of fluid to be removed during HD, specifically the ultrafiltration volume (UV). Increased ultrafiltration rate is linked to unfavourable cardiovascular outcomes [10,11].

Fluid overload, together with conventional and end-stage kidney disease-related risk factors, like renal anemia, chronic inflammation, vascular calcifications, secondary hyperparathyroidism, etc., leads to structural and functional changes in the myocardium and the arteries. We have previously described the relationship between UV variability and myocardial abnormalities in chronic dialysis patients [12]. We hypothesize that changes in the periodic volume loading of the myocardium can significantly contribute to worse patient outcomes.

Visit-to-visit UV variability has also been described to predict all-cause mortality in Chinese patients on chronic hemodialysis [13].

We do not have much data on the effect of visit-to-visit UV variability on CV mortality and CV events in HD chronic patients. Therefore, our study’s goal was to evaluate this effect.

## 2. Materials and Methods

### 2.1. Study Subjects

**Figure 1:**
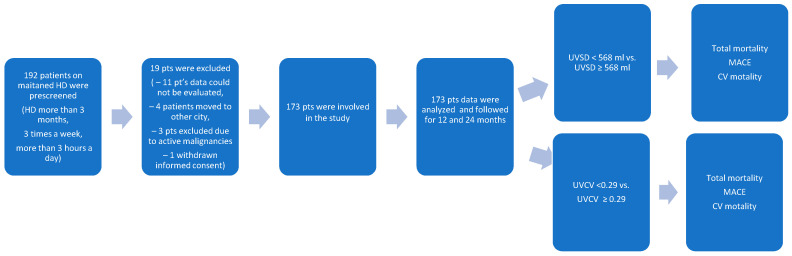
The flowchart shows the procedure for selecting study participants.

Data of 173 HD patients dialyzed from January 2022 to December 2023 at the National Dialysis Center Pécs were retrospectively analyzed (Figure 1). Patients on maintenance HD treatment in our center for at least 3 months were included. All patients received 4 h of conventional HD three times weekly. The sodium concentration of the dialysis fluid was 135–140 mmol/L, individualized if needed, but preferably unchanged during the observation period. The observation period lasted until the occurrence of a major adverse cardiovascular event (MACE) or death. The most recent visit was the last follow-up time, otherwise. The inclusion criteria were as follows: patients received maintenance HD treatment in our center for at least 3 months, and Kt/V was ≥ 1.2. The exclusion criteria were as follows: age < 18 years; temporary dialysis in our center; kidney transplantation during the observation period; having less than 3 HD sessions/week; and having an unclear medical history or missing clinical information (Table 1). Clinical and laboratory data; age; sex; body mass index (BMI); the presence of hypertension, diabetes, and cardiovascular disease (myocardial infarction, cerebral infarction, cerebral hemorrhage, coronary heart disease, chronic heart failure, and peripheral vascular diseases); and the etiology of chronic kidney disease were all recorded before the beginning of the observation period.

Blood pressure measurements were performed pre-HD, every 60 min during the HD treatment, and post-HD with a calibrated sphygmomanometer. Serum levels of intact parathyroid hormone (iPTH), albumin, calcium, phosphorus, and hemoglobin were obtained from the most recent regular laboratory examinations using blood samples collected before HD.

### 2.2. Definitions of UV Variability

After a dialysis adaptation period lasting at least 3 months, the observation period started: a series of visits were conducted for 12 consecutive months to evaluate UV variability. Every dialysis was regarded as a visit where dialysis-related information was recorded. The UV variability indices were utilized to compute the coefficient of variation (CV) and standard deviation (SD). Dividing the SD by the mean yields the CV.

UV data during the observation period were recorded to calculate UV variability [13].

For the calculations, the following formulas were used:

$$\mathrm{UVSD}=\frac{\sqrt{\sum_{i=1}^{n}{{(\mathrm{UV}}_{i}\overset{-}{-\mathrm{UV})}}^{2}}}{n-1}\;\;\mathrm{UVCV}=\frac{\mathrm{UVSD}}{\mathrm{mean}}$$

where *n* is each patient’s total dialysis number, and the mean was determined using all UV data.

Definition of major adverse cardiovascular events:

Major adverse cardiovascular events (MACEs) were defined as a composite endpoint of nonfatal stroke, nonfatal myocardial infarction, peripheral artery revascularization, and cardiovascular death.

### 2.3. Conventional Echocardiography Measurement

Comprehensive echocardiographic measurements were conducted using a 3 MHz transducer and a commercial ultrasound system (Mindray DC-80A, Mindray Global, Nanshan, Shenzhen, China). Standard imaging methods, such as M-mode, two-dimensional, and Doppler measurements, were used. Transmitral inflow velocities were assessed using a pulsed-wave Doppler in the apical four-chamber view, with the sample volume placed at the tips of the mitral valve leaflet. Early diastolic (E-wave) velocities were measured. LAD was determined using 2D-guided M-mode echocardiography in the parasternal short-axis view at the base of the heart.

Left atrial (LA) volume was computed using the area-length approximation formula: LA volume (mL) = [8/(3π)] [(A1 × A2)/L], where A1 and A2 represent the corresponding LA areas measured in the apical two- and four-chamber views. Of the two long axes measured in each perspective, the LA length is the shortest. The following formula was used to obtain the LV mass: IVS stands for the interventricular septum. LV mass (g) = 0.8 × {1.04 × ([IVS + LVID + PWT]^3^ − [LVID]^3^)} + 0.6, LVID is the LV internal diameter, and PWT denotes the inferolateral wall thickness. All measurements were obtained at end-diastole. To correct for BSA, the LVMI was calculated by dividing LV mass by BSA. We measured LVEDV and LVEF with the biplane-modified Simpson’s rule. The echocardiogram was carried out by qualified cardiologists who were blind to the clinical data about the patients.

### 2.4. Statistical Analysis

Continuous variables are shown as the average ± SD or the median and interquartile range depending on the normal distribution results, using the Kolmogorov–Smirnov test, whereas categorical variables are described as percentages. For normally distributed data, a *t*-test was used; for non-normally distributed data, the Mann–Whitney U test was used.

A chi-squared test was applied to variable data. Analysis of variance (ANOVA) was conducted to evaluate differences between study subgroups. Odds ratios (ORs) were estimated using regression analyses for the relationships between all covariates (including demographic, clinical, and laboratory information), and logistic regression analysis was made to evaluate all-cause mortality and MACE’s influencing factors. The Kaplan–Meier method was used to display the connection between UV variability and all-cause mortality, and the log-rank test was used to evaluate the results.

Survival analysis was used to compare the efficiency of two UV variability indices in predicting 1- and 2-year survival rates. All statistical analyses were performed with SPSS Statistics 23 (IBM Company, Chicago, IL, USA). For multiple comparisons, Bonferroni’s correction was applied, and *p* < 0.05 was considered significant.

## 3. Results

The patient recruitment chart is shown in Figure 1. A total of 192 patients were prescreened. In total, 19 patients were excluded: 11 patients had missing data, 4 patients moved to other cities, 3 patients had active malignancy, and 1 patient withdrew his consent. Thus, a total of 173 patients were enrolled in the study.

Baseline characteristics and laboratory data of the participating patients are shown in Table 2. In the final analysis, 173 patients were included. The average age was 63 ± 13 years, with 53% of them being male. A total of 36 (21%) participants had diabetes, and 42 (24%) had CV disease. In 73% of the patients, arteriovenous fistulas were used for dialysis. CKD etiology was the following: 19 (11%) patients had glomerulonephritis, 34 (20%) had diabetic nephropathy, 82 (47%) had hypertension, 20 (12%) had polycystic kidney disease, and 18 (10%) had other causes.

The high- and low-UVSD and -UVCV group’s all-cause mortality, CV mortality, and MACE event rates are shown in Table 3. All-cause mortality was significantly higher in the high-UVSD group compared to the low-UVSD group after 12 months (11/84 vs. 4/89; *p* = 0.022) and after 24 months (21/84 vs. 9/89; *p* < 0.001). Similarly, mortality was higher in the high-UVCV group compared to the low-UVCV group after 12 months (9/78 vs. 6/95; *p* = 0.045) and after 24 months (18/78 vs. 12/95; *p* = 0.005). Major adverse CV event (MACE) rates were also significantly higher in the high-UVSD group compared to the low-UVSD group (10/84 vs. 4/89; *p* = 0.037) after 12 months and (20/84 vs. 8/89; *p* < 0.001) after 24 months, respectively. Similarly, the MACE rate was significantly higher in the high-UVCV group compared to the low-UVCV group after 12 months (8/78 vs. 5/95; *p* = 0.046) and after 24 months (15/78 vs. 13/95; *p* = 0.029) in the follow-up, respectively.

The median value of UVSD was 568.0 mL. Based on the median value of UVSD, patients were divided into two groups: higher- (≥568) and lower UVSD (<568) groups. The median value of UVCV was 0.29. Based on the median value of UVCV, patients were divided again into two groups: higher- (≥0.29) and lower UVCV (<0.29) groups. There was no difference in dialysis vintage between the groups (Table 2).

Compared with lower UVSD patients, those having higher UVSD were older, more likely to be male, and more likely to be on erythropoietin therapy. They had significantly higher levels of serum phosphorus, iPTH, and CRP. Left ventricular mass index (LVMI) was higher in the high-UVSD group than in the low-UVSD group (137.63 vs. 147.8; *p* = 0.019) and higher in the high-UVCV group (133.83 vs. 147.44; *p* = 0.023). Left ventricular end-diastolic diameter (LVEDD) and end-systolic diameter (LVESD) were higher in the high-UVCV group compared to the low-UVCV group (49.95 vs. 52.08; 0.013 and 32.19 vs. 34.13; *p* = 0.034). Also, the right and left atrial volumes (RAV and LAV) were significantly higher in the high-UVCV group (43.32 vs. 63.81; *p* = 0.001 and 50.76 vs. 73.6; *p* < 0.001). The average ultrafiltration volume was not different between the compared groups (Table 2).

Patients with high UVCV had higher hemoglobin levels than those with low UVCV despite the less frequent use of erythropoietin in this group. The average ultrafiltration volume was lower in patients with higher UVCV.

LVMI, LVEDD, LVSD, RAV, and LAV were higher in the high-UVCV group compared with the low-UVCV group (Table 2).

**Figure 2:**
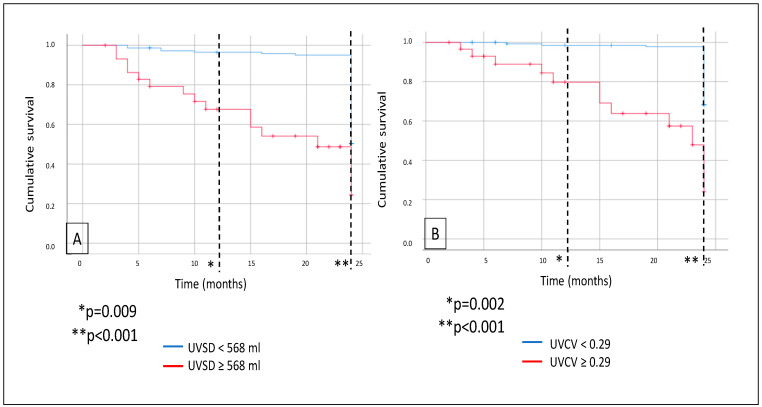
In hemodialysis patients, 12- and 24-month all-cause mortality (**A**) in case of UVSD <568 mL vs. ≥568 mL; and 12- and 24-month all-cause mortality (**B**) in case of UVCV <0.29 vs. ≥0.29.

Mortality was analyzed: during a median follow-up of 24 months, a total of 30 patients died. As demonstrated on the Kaplan–Meier curves, all-cause mortality was significantly higher in the high-UVSD group compared to the low-UVSD group after 12 months (*p* = 0.009) and even more after 24 months of follow-up (*p* < 0.001). Similarly, all-cause mortality was higher in the high-UVCV group than in the low-UVCV group after 12 months (*p* = 0.002) and even more after 24 months of follow-up (*p* < 0.001) (Figure 2).

**Figure 3:**
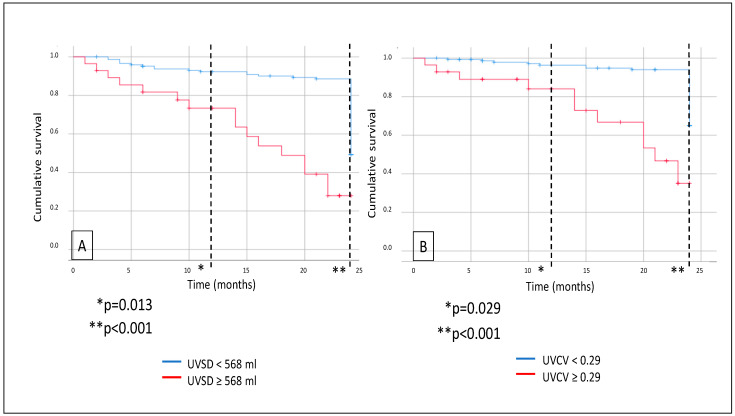
In hemodialysis patients, 12- and 24-month MACE events (**A**) in case of UVSD <568 mL vs. ≥568 mL; and 12- and 24-month all-cause mortality (**B**) in case of UVCV <0.29 vs. ≥0.29.

Further survival analysis revealed that major adverse CV event (MACE) rates were also significantly higher in the high-UVSD group than in the low-UVSD group after 12 months (*p* = 0.013), and the difference was even higher after 24 months of follow-up (*p* < 0.001). High-UVCV patients had higher MACE rates than low-UVCV patients after 12 months (*p* = 0.029) and 24 months of follow-up (*p* < 0.001), with the difference increasing over time (Figure 3). Still, we did not find any significant difference in CV mortality between the low- and high-UVSD and -UVCV groups.

**Figure 4:**
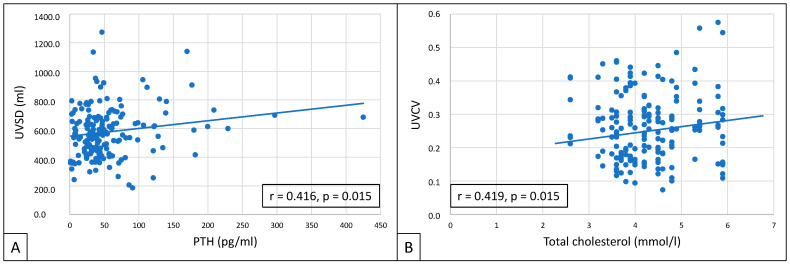
Correlations between UVSD and PTH (**A**), and between UVCV and total cholesterol (**B**).

UVSD showed a close correlation with PTH (r = 0.416; *p* = 0.015), and UVCV with total cholesterol (r = 0.419; *p* = 0.015) (Figure 4).

Multivariate analyses were performed on clinical data, metabolic parameters, ultrafiltration parameters, echocardiographic parameters, and laboratory results. The significant confounding factors of UVSD were age (OR = 1.295, *p* = 0.019), gender (OR = 1.361, *p* = 0.046), BMI (OR = 1.288, *p* = 0.045), phosphate (OR = 1.691; *p* = 0.011), and PTH (OR =1.698; *p* = 0.004). The significant confounding factors of UVCV were gender (OR = 1.153, *p* = 0.024), HT (OR = 1.407, *p* = 0.001), LVMI (OR = 1.602, *p* = 0.003), LVEDD (OR = 1.211, *p* = 0.028), and serum albumin (OR = 1.126, *p* = 0.005) (Table 4).

Logistic regression analysis showed that MACE-independent predictors were UVSD, DM, and CRP, and all-cause mortality’s independent predictors were USD and UVCV (Table 5).

## 4. Discussion

The present study demonstrated that visit-to-visit UV variability parameters, UVSD and UVCV, could predict all-cause mortality and MACEs but not CV mortality in patients with chronic HD. Although we could not clarify the underlying causes, our data were in line with and also extended some previous observations.

As ESRD patients’ kidneys are unable to maintain fluid balance, volume overload is common in HD patients [14]. Excessive interdialytic weight gain (IDWG) and the consequent excessive intradialytic weight loss constitute cyclical, multiple-system organ stress in the cardiovascular system [15]. Previous studies have shown that higher IDWG results in cardiac chamber remodeling [16], diastolic dysfunction, and myocardial stunning [17,18]. The volume fluctuation also has detrimental effects on the peripheral arteries [19,20]. These cardiovascular injuries are associated with increased all-cause and cardiovascular mortality [21,22,23]. It has also been documented that a high ultrafiltration rate is associated with increased mortality in HD patients [24,25,26].

The novelty of our study is that not only a high UF rate or volume but also a high visit-to-visit UV variability may increase the number of MACEs and may increase all-cause mortality. MACE and all-cause death rates were greater in patients with high UVSD than in those with low UVSD, even though their UF rates were comparable. Similarly, patients with high UVCV had higher all-cause mortality and MACE rates than those with low UVCV. The differences between the groups were significant after 12 months and were even more pronounced after 2 years. High UF volume variability may have a different effect on the cardiac remodeling than the high UF volume per se: in our study, the only difference in the measured echocardiographic parameters between the high- and low-UVSD groups was the higher LVMI in the previous group.

In the high-UVCV group, the average single HD UF volume was lower than in the low-UVCV group. As expected, in this group with a lower UF volume, LVMI, LVEDD, LVESD, RAV, and LAV were also lower, indicating less cardiac remodeling than in the other group. Interestingly, UVCV, a marker of the UV variability, seemed to increase the all-cause mortality and MACE rate even though the UF volume was lower, but the echocardiographic abnormalities were more pronounced in the high-UVCV group, suggesting that, eventually, UF variability may be a more important predictor of survival and MACE than UF volume per se in HD patients’ developing cardiovascular disease, at least in certain phases.

According to our results, all-cause mortality and MACE were significantly increased, but there was no difference in cardiovascular mortality between the high- and low-UVSD and -UVCV groups, which can be explained by the small sample size or endpoint definition bias (e.g., high rate of non-cardiovascular deaths) and the short follow-up time.

In patients with high UVSD, we have seen a clustering of traditional and CKD-related cardiovascular risk factors: these patients were older, were more likely to be male, had higher phosphate and PTH levels, and had higher LMVI than patients with lower UVSD. A high PTH level, a well-known cardiovascular risk factor in dialysis patients [27,28], has shown a good correlation with UVSD. The abovementioned factors might also affect the worse survival and higher MACE rate seen in high-UVSD patients. In high-UVCV patients, we have seen a more pronounced clustering of classic or emerging cardiovascular risk factors, and these patients also have worse survival and a higher MACE rate than those having low UVCV. High-UVCV patients had higher LVMI, LVEDD, LVESD, RAV, and LAV, and the average UF volume was also less than in the other group. UVCV has also shown a good correlation with a classical cardiovascular risk factor, total cholesterol level [29].

Although both UVSD and UVCV are measures of UF variability, and, according to our results, both of them are linked to survival and MACE occurrence, they are in some way different measures of variability and do have different clustering with known cardiovascular risk factors. High UF variability is causing increased shear stress on the arterial walls and may increase the activation of the sympathetic nervous system. It can precipitate endothelial response, leading to ischemic damage of the target organs and eventually to vascular calcifications and accelerated atherosclerosis [30]. We can assume that another explanation for the worse outcome could be the cumulative impairment of periodic volume loading, which could be a possible pathomechanism of the development of heart failure in ESRD patients on regular hemodialysis. This pathophysiological change may be one piece of the puzzle of uremic cardiomyopathy.

Consequently, a higher risk of cardiovascular function impairment and unfavourable outcomes is associated with increased UV variability. From this perspective, the link between higher UV variability and increased all-cause mortality seems to be reasonable and plausible. Moreover, the variability of other physiological parameters in HD patients has also been studied. High variability of physiological parameters has generally been associated with poor cardiovascular outcomes. Numerous studies have shown that high blood pressure variability is a risk factor for short- and long-term cardiovascular and all-cause mortality in dialysis patients [31,32,33,34]. It has been hypothesized that volume losses occurring with dialysis induce baroreceptor-dependent changes in autonomic function that stabilize venous return (capacitance vessel constriction) and peripheral arteriolar constriction. Subjects with inelastic conduit vessels, like HD patients, cannot adequately sense the fluctuations in blood pressure and do not mount an effective counter-regulatory response [35,36,37]. Intravascular volume variability may have a similar effect to blood pressure variability on the same receptors, explaining the poor outcome in patients having high UVSD or UVCV. Additionally, several other studies reported that high variability of serum phosphorus [38], serum albumin [39], hemoglobin [40], and heart rate [41] could also predict mortality in HD patients. Together with the findings mentioned above, our data on increased UV variability provide evidence that high variability of several physiological parameters may be linked to unfavourable clinical outcomes. The exact mechanisms behind these relationships remain unknown and require more research.

Studies investigating risk factors for mortality in the HD population have revealed that patients who are male and older with preexisting CVD and DM had a higher risk of cardiac death [42,43,44,45]. Zhang et al. demonstrated that high UV variability, especially UVCV, can predict all-cause mortality in patients receiving HD, especially for older patients, males, and those with comorbidities [13]. This may be attributed to the preexistence of or predisposition to cardiovascular diseases in such populations.

Han et al. have investigated whether fluid overload and its potential interaction affect the relationship between vascular stiffness and LVDD in dialysis-naive patients with stage 5 CKD. According to their results, fluid overload can be an important factor aggravating LVDD in patients with CKD having increased arterial stiffness. It is advisable to conduct simultaneous assessments of vascular stiffness, fluid balance, and LV function, particularly in the specific groups mentioned earlier [46].

One of the pathogenetic processes of LVDD in the general population is left ventricular hypertrophy (LVH). Both LVDD and LVH are prevalent and strongly associated with survival in CKD patients [47]. Morphological and functional alterations of the heart and the arteries do affect CV events and survival [48] and may be linked to high UF variability in HD patients.

The present study has several limitations. It is an observational study with a relatively small sample size. The follow-up period of the study was too short to predict accurate long-term survival, and the number of CV deaths was also relatively small.

## 5. Conclusions

Our study demonstrates that high UVSD and UVCD are associated with increased all-cause mortality and MACE rates but not CV mortality in chronic HD patients. Cardiovascular changes caused by increased UF volume variability during HD may contribute to higher CV morbidity and mortality in these patients.

## Figures and Tables

**Table 1:** Inclusion and exclusion criteria of the study.

Inclusion Criteria	Exclusion Criteria
Age ≥ 18 years	Any unstable condition
On regular hemodialysis, three times a week for >3 months	Having less than 3 HD sessions/week
With a single-pool Kt/V ≥ 1.2	Active infection or malignancy
	Pregnancy or lactating
	Known alcohol or drug abuse
	Immunosuppressive drug use
	Major visual or hearing impairments
	Kidney transplantation during the observation period
	Having unclear medical history or missing clinical information.

**Table 2:** Baseline clinical data.

Clinical Data	HD Patients (n = 173)	UVSD < 568 mL (n = 89)	UVSD ≥ 568 mL (n = 84)	*p*	UVCV < 0.29 (n = 95)	UVCV ≥ 0.29 (n = 78)	*p*/NS
Man/woman (n/%)	91/82 (53/47)	38/51 (43/57)	53/31 (63/37)	0.003 *	57/38 (60/40)	33/45 (42/58)	0.01 *
Age (year)	63 ± 13	59 ± 12.5	67 ± 12.5	0.001 *	64 ± 13.4	62 ± 12.3	NS
Duration of kidney disease (year)	10.8 ± 9.4	11.5 ± 10	10 ± 9	NS	11.2 ± 10	10.4 ± 9	NS
Dialysis vintage (months)	224 ± 34	168 ± 30	143 ± 27	NS	165 ± 28	145 ± 27	NS
EPO usage (n/%)	108 (62)	45 (53)	63 (71)	0.009 *	69 (73)	38 (49)	0.003 *
Metabolic parameters							
Hypertension (n, %)	168 (97)	82 (98)	86 (97)	NS	93 (98)	75 (96)	NS
BMI (kg/m^2^)	27.7 ± 4.6	26.9 ± 4.5	28.5 ± 4.7	NS	27.8 ± 5.8	27.5 ± 6.3	NS
Dyslipidemia (n, %)	72 (42)	36 (43)	38 (43)	NS	34 (36)	38 (49)	NS
Diabetes (n, %)	36 (21)	17 (20)	19 (21)	NS	23 (24)	13 (17)	NS
Ultrafiltration parameters							
Ultrafiltration volume (single HD)	2355.66 ± 718.26	2331.58 ± 826.65	2381.19 ± 580.7	NS	2681.62 ± 626.6	1854.64 ± 547.7	0.01
Echocardiographic parameters							
LVEF (%)	56.61 ± 8.81	57.04 ± 7.78	56.19 ± 9.7	NS	56.06 ± 8.9	57.5 ± 8.5	NS
LVMI (g/m^2^)	142.62 ± 39.36	137.63 ± 37.43	147.8 ± 40.67	0.019 *	133.83 ± 42.5	147.44 ± 36.2	0.023 *
LVM (g)	253.70 (129.3–418.1)	259.2 (137.25–427.42)	253.39 (129.33–420.2)	NS	248.18 (119.3–395.61)	266.94 (147.83–556.99)	NS
LVEDD (mm)	51.24 ± 5.8	50.96 ± 5.95	51.53 ± 5.64	NS	49.95 ± 5.99	52.08 ± 5.54	0.013 *
LVESD (mm)	32 (20–56)	33 (22–55)	31 (20–56)	NS	30 (20–50)	34 (23–56)	0.034 *
E/A	0.78 (0.34–2.3)	0.75 (0.35–2.21)	0.83 (0.36–2.30)	NS	0.79 (0.35–2.20)	0.79 (0.36–1.69)	NS
DD (n/%)	94 (54)	44 (52)	50 (56)	NS	51 (54)	42 (54)	NS
RAV (mL/m^2^)	45.3 (10.4–138.7)	42.6 (10.42–109.54)	45.2 (18.04–118.13)	NS	49.92 (14.94–118.14)	41.6 (10.42–95.76)	0.001 *
LAV (mL/m^2^)	54.6 (15.2–115.0)	53.0 (16.03–155.01)	55.94 (15.2–125.75)	NS	50.85 (15.2–112.53)	54.08 (20.69–115.01)	<0.001 *
RVP (mmHg)	33.44 ± 8.2	33.48 ± 7.44	33.4 ± 8.91	NS	34.04 ± 8.32	32.71 ± 8.0	NS
Laboratory results							
Hb (g/dL)	13.6 ± 1.53	13.6 ± 1.54	13.7 ± 1.56	NS	10.84 ± 1.04	11.26 ± 1.38	0.012 *
TP (g/L)	64.36 ± 4.97	63.25 ± 4.76	65.54 ± 4.91	NS	63.93 ± 4.65	65.03 ± 5.39	NS
Albumin (g/L)	39.3 (15.4–45.3)	40.1 (31.8–44.8)	39.7 (15.4–45.3)	NS	40.3 (33.3–44.4)	39.3 (15.5–45.3)	NS
Ca (mmol/L)	2.22 ± 0.18	2.21 ± 0.16	2.42 ± 0.19	NS	2.22 ± 0.18	2.24 ± 0.17	NS
P (mmol/L)	1.68 (0.34–3.1)	1.56 (0.77–2.40)	1.87 (0.95–3.50)	<0.001 *	1.69 (0.77–3.23)	1.75 (0.92–2.4)	NS
PTH (pg/mL)	42.6 (1.4–297)	37 (1.40–182)	46.9 (3.09–297)	0.027 *	46.1 (1.4–229)	39.5 (3.09–297)	NS
CRP (mg/L)	4.6 (3.3–16.68)	3.5 (0.30–16.10)	5.8 (0.30–52.10)	0.033 *	4.1 (0.40–16.10)	5.10 (0.30–42.60)	NS
Creatinine (umol/L)	856.98 ± 184.3	832.22 ± 165.8	976.12 ± 205.04	NS	849.1 ± 187.6	897.3 ± 199.34	NS
Cardiovascular disease in the history							
Total	42 (24)	20 (24)	22 (25)	NS	18 (19)	24 (31)	NS
Myocardial infarction (n, %)	10 (6)	4 (5)	6 (7)	NS	5 (5)	5 (6)	NS
Stroke (n, %)	8 (5)	3 (4)	5 (6)	NS	3 (3)	5 (6)	NS
Peripheral artery disease (n, %)	12 (7)	4 (5)	8 (9)	NS	5 (5)	7 (9)	NS
Revascularization (n, %)	12 (7)	4 (5)	8 (9)	NS	5 (5)	7 (9)	NS
Kidney disease							
Hypertensive nephropathy (n, %)	82 (47)	45 (53)	35 (39)	0.043 *	48 (50)	34 (43)	NS
Diabetic nephropathy (n, %)	34 (20)	15 (18)	19 (21)	NS	19 (20)	15 (19)	NS
Glomerulonephritis (n, %)	19 (11)	4 (5)	13 (14)	0.040 *	14 (15)	5 (6)	0.045 *
ADPKD (n, %)	20 (11)	9 (11)	8 (9)	NS	10 (10)	10 (13)	NS
Other (n, %)	18 10)	11 (13)	14 (16)	NS	16 (17)	2 (2)	0.01 *

* *p* < 0.05. NS, nonsignificant; EPO, erythropoietin; BMI, body mass index; UVSD, ultrafiltration volume standard deviation; UVCV, ultrafiltration volume coefficient of variation; HD, hemodialysis; LVEF, left ventricular ejection fraction; LVMI, left ventricular mass index; LVM, left ventricular mass; LVEDD, left ventricular end-diastolic diameter; LVESD, left ventricular end-systolic diameter; E/A, early/late wave of the mitral inflow; DD, diastolic dysfunction; RAV, right atrial volume; LAV, left atrial volume; RVP, right ventricular pressure; Hb, hemoglobin; TP, serum total protein; Ca, calcium; P, phosphorous; PTH, parathormone; CRP, C-reactive protein; ADPKD, autosomal dominant polycystic kidney disease.

**Table 3:** All-cause mortality, CV mortality, and MACE rates in different UVSD and UVCV groups.

	Total HD Patients (n = 173)	UVSD High (≥568 mL) (n = 84)	UVSD Low (<568 mL) (n = 89)	UVCV High (≥0.29) (n = 78)	UVSD Low (<0.29) (n = 95)
After 12 months of follow-up					
All-cause mortality events (n/%)	15 (9)	11 (13)	4 (4)	9 (11)	6 (6)
CV mortality events (n/%)	7 (4)	4 (5)	3 (3)	4 (5)	3 (3)
MACE (n/%)	14 (8)	10 (12)	4 (4)	9 (11)	5 (5)
After 24 months of follow-up					
All-cause mortality events (n/%)	30 (17)	21 (25)	9 (10)	18 (23)	12 (12.6)
CV mortality events (n/%)	17 (9.8)	10 (12)	7 (7.8)	10 (13)	7 (7)
MACE (n/%)	28 (16)	20 (24)	8 (9)	15 (17)	13 (13.6)

CV, cardiovascular; MACE, major adverse cardiovascular event; HD, hemodialysis; UVSD, ultrafiltration volume variability standard deviation; UVCV, ultrafiltration volume coefficient variability.

**Table 4:** Multivariate regression analysis.

	UVSD				UVCV			
	B	Std. Errors	Confidence Interval 95%	*p*	B	Std. Errors	Confidence Interval 95%	*p*
Age	1.295	0.104	1.090–1.490	* 0.019	0.251	0.013	0.219–1.283	0.287
Gender	1.361	0.146	1.193–1.639	* 0.046	1.153	0.024	1.283–1.584	* 0.024
BMI	1.288	0.017	1.094–1.483	* 0.045	0.527	0.436	0.106–1.948	0.544
HT	0.332	0.076	0.025–2.740	0.066	7.469	1.407	3.366–10.738	* 0.001
LVMI	0.871	0.191	0.816–1.075	0.155	1.602	0.225	1.218–1.782	* 0.003
LVEDD	0.473	0.354	0.131–1.809	0.253	1.211	0.079	1.182–1.318	* 0.028
E/A	0.860	0.176	0.678–1.959	0.057	0.374	0.166	0.204–1.482	0.420
CRP	0.732	0.145	0.486–1.949	0.703	0.498	0.198	0.257–1.526	0.471
P	1.691	0.117	1.192–1.837	* 0.011	0.050	0.011	0.031–1.071	0.733
Albumin	0.258	0.192	0.205–1.991	0.822	1.126	0.102	1.102–2.331	* 0.005
PTH	1.698	0.258	1.389–1.890	* 0.004	0.030	0.027	0.024–0.084	0.279

* *p* < 0.05. UVSD, ultrafiltration volume standard deviation; UVCV, ultrafiltration volume coefficient of variation; HT, hypertension; BMI, body mass index; LVMI, left ventricular myocardial mass index; LVEDD, left ventricular end-diastolic diameter; E/A, early and late wave of mitral inflow; CRP, C-reactive protein; P, serum phosphate; PTH, parathormone.

**Table 5:** Logistic regression analysis of all-cause mortality and MACE.

All-Cause Mortality (r = 0.390, r^2^ = 0.153)	Exp (B) (OR)	95% CI for Exp (B) Lower–Upper	*p*
UVSD	1.108	1.001–1.191	* 0.046
UVCV	1.394	1.278–1.568	* 0.042
Gender	0.074	0.047–1.115	0.675
HT	0.146	0.033–2.292	0.344
DM	0.076	0.067–3.231	0.201
BMI	0.007	0.004–1.017	0.466
RVP	0.547	0.137–1.812	0.061
E/A	0.051	0.037–1.094	0.760
Hb	0.033	0.010–1.051	0.598
CRP	0.002	0.001–1.008	0.320
Albumin	0.007	0.005–1.011	0.254
PTH	0.002	0.001–1.781	0.740
**MACE** **(r = 0.473, r^2^ = 0.224)**	**Exp (B)**	**95% CI for Exp (B)** **Lower-Upper**	***p***
UVSD	0.747	0.269–1.867	0.083
UVCV	2.160	1.340–2.256	* 0.033
Gender	0.011	0.065–1.130	0.478
HT	0.214	0.005–1.439	0.289
DM	1.277	1.060–2.494	* 0.001
BMI	0.011	0.006–1.013	0.641
RVP	0.687	0.183–1.220	0.095
E/A	0.092	0.022–1.147	0.591
Hb	0.059	0.030–1.198	0.122
CRP	1.220	1.145–1.254	* 0.011
Albumin	0.013	0.003–1.144	0.196
PTH	1.005	1.003–1.255	* 0.003

* *p* < 0.05. UVSD, ultrafiltration volume standard deviation; UVCV, ultrafiltration volume coefficient of variation; HT, hypertension; DM, diabetes mellitus; BMI, body mass index; RVP, right ventricular pressure; E/A, early and late wave of mitral inflow; Hb, hemoglobin; CRP, C-reactive protein; PTH, parathormone.

## Data Availability

The data underlying this article cannot be shared publicly due to Hungarian regulations and the privacy of individuals who participated in the study. The data could be shared upon reasonable request made to the corresponding author if accepted by the Regional Committee for Medical and Health Research Ethics and local Data Protection Officials.
